# Methylation of a panel of genes in peripheral blood leukocytes is associated with colorectal cancer

**DOI:** 10.1038/srep29922

**Published:** 2016-07-25

**Authors:** Xiang Luo, Rong Huang, Hongru Sun, Yupeng Liu, Haoran Bi, Jing Li, Hongyuan Yu, Jiamei Sun, Shangqun Lin, Binbin Cui, Yashuang Zhao

**Affiliations:** 1 Department of Epidemiology, Public Health College, Harbin Medical University, 157 Baojian Street, Harbin, Heilongjiang, 150006, People’s Republic of China; 2 Department of Gastrointestinal Surgery, Cancer Hospital of Harbin Medical University, 150 Haping Street, Harbin, Heilongjiang, 150006, People’s Republic of China

## Abstract

The relationship between the DNA methylation status of the CpG islands of multiple genes in blood leukocytes in CRC susceptibility and prognosis, as well as possible interactions with dietary factors on CRC risk are unclear. We carried out a case-control study including 421 CRC patients and 506 controls to examine the associations between six genes (*AOX-1*, *RARB2*, *RERG*, *ADAMTS9*, *IRF4*, and *FOXE-1*), multiple CpG site methylation (MCSM) and susceptibility to CRC. High-level MCSM (MCSM-H) was defined as methylation of greater than or equal to 2 of 5 candidate genes (except for *RARB2*); low-level MCSM (MCSM-L) was when 1 candidate gene was methylated; non-MCSM was when none of the candidate genes were methylated. Blood cell-derived DNA methylation status was detected using methylation-sensitive high-resolution melting analysis. The hypermethylation status of each individual gene was statistically significantly associated with CRC. MCSM status was also associated with CRC (OR = 1.54, 95% CI: 1.15–2.05, *P* = 0.004). We observed interactions between a high level of dietary intake of cereals, pungent food, and stewed fish with brown sauce, age (older than 60 yrs), smoking and hypermethylation on risk of CRC. MCSM in peripheral blood DNA may be an important biomarker for susceptibility to CRC.

The development of colorectal cancer (CRC) is characterized by genetic and epigenetic dysregulation of signal transduction cascades in a multi-step fashion. The long-term accumulation of epigenetic changes can lead to the occurrence of CRC^1^. DNA methylation, a major epigenetic modification, induces transcriptional silencing of tumor suppressor genes^2^ and the subsequent expression of disease phenotypes without any changes to the primary DNA sequences^3^, which has been identified as a critical step in tumor initiation^4^, including in CRC. Genes known to be methylated that are detected and studied in tumor-derived DNA in colorectal cancer are *CDKN2A*^5^, *CDH4*^6^, *NEUROG1*^7^ and *MLH1*^8^,^9^ and we tested a gene panel consisting of *APC*, *MGMT, RASSF2A* and *WIF-1*^10^ in our previous study. As a follow-up study, we then searched for genes with aberrant promoter hypermethylation associated with CRC in the PubMeth^11^, MethDB^12^ and MethyCancer^13^ literature databases. It has been reported that gene-specific hypermethylation changes in promoter CpG islands are related to biological processes of tumor progression including metastasis suppressors (*AOX-1*^14^ and *RARB2*^15^), angiogenesis inhibitors (*RERG*^16^ and *ADAMTS9*^17^) and signal transcription factors (*IRF4*^18^ and *FOXE-1*^19^).

RAS-like estrogen-regulated growth inhibitor (*RERG*) was first detected in breast cancer using microarray analysis^16^, after which significantly hypermethylated *RERG* was observed in colorectal adenocarcinomas but not in adenomas and normal mucosa^20^. Aldehyde oxidase 1 (*AOX-1*) was found to be down-regulated and hypermethylated in CRC tissues, suggesting a potential functional role of this gene in cancer development^21^. *ADAM* metallopeptidase with thrombospondin type 1 motif, 9 (*ADAMTS9*) which has been characterized as a novel tumor suppressor gene, has been epigenetically silenced in lymph node metastases in nasopharyngeal carcinoma^17^. Interferon regulatory factor 4 (*IRF4*) was found to be highly silenced by DNA hypermethylation in both gastric cancers and gastric mucosa from cancer patients, which suggested that *IRF4* hypermethylation may be a useful molecular marker for diagnosing recurring gastric cancer^22^. Hypermethylation of forkhead box E1 (*FOXE1*) has also been explored as a noninvasive biomarker for the detection of CRC in stool samples^19^. Finally, retinoic acid receptor beta 2 (*RARB2*) was frequently inactivated in cancers of epithelial origin and has been shown to have tumor suppressive activity in lung, breast, and colon cancer cell lines^15^. In addition, promoter hypermethylation of *RARB2* was found in stool samples (13%) of patients with inflammatory bowel disease, but with no aberrant hypermethylation in healthy subjects^23^.

Recently, researchers have frequently focused on tumor tissues to explore the relationship between DNA methylation status and CRC as potential biomarkers^17^,^19^,^20^,^24^,^25^. Furthermore, studies have shown that dietary habits and lifestyle habits, such as drinking and smoking may impact DNA methylation^26^,^27^,^28^. In addition, tumors do not only develop as an isolated phenomenon in their target tissues^29^ and emerging data suggests that leukocyte DNA methylation might be linked to susceptibility to CRC^30^. CpG island methylation phenotypes (CIMP) were first introduced by Toyota *et al*. to define extensive hypermethylation of multiple gene CpG islands from tumor tissues, which is currently recognized as one of the major mechanisms in CRC carcinogenesis^31^,^32^. Multiple genetic and epigenetic alterations that contribute to chemoresistance occur during chemotherapy and may eventually impact the disease outcome^33^. Therefore, we propose that the DNA methylation status of multiple gene CpG islands from blood leukocytes may be associated with the risk and prognosis of CRC. Thus, we carried out this study to explore the interaction between environmental factors and DNA methylation status on the risk of CRC.

## Results

### Characteristics of the study subjects

This study consisted of 421 cases (163 females and 258 males) with a mean age of 59.46 ± 11.41 and 506 controls (227 females and 279 males) with a mean age of 56.62 ± 10.97 (*P* = 0.000; Table 1). The proportion of age in groups of 60 yrs to 69 yrs and greater than 70 yrs in cases were both higher than those in controls. The proportion of overweight (BMI ≥ 23.0) in controls (63.4%) was higher than that in cases (52.4%; *P* = 0.003). The distribution of different occupational categories and family history of other cancers were also different between cases and controls (*P* = 0.021 and *P* = 0.004, respectively). The proportion of mental workers in controls (52.9%) was higher than that in cases (43.9%). The proportion of patients with family history of other cancers in cases (78.4%) was lower than that in controls (86.5%).

### Association between the methylation status of six individual genes and CRC

Statistically significant associations between the methylation of *AOX-1*, *RERG*, *ADAMTS9*, *IRF4*, *FOXE-1* and CRC were observed in univariate logistic regression analyses (Table 2). Further multivariate logistic regression analyses with adjustment for age, BMI, occupation and family history of cancer showed that the hypermethylation of *AOX-1* (OR = 1.72, 95% CI: 1.30–2.27, *P* = 0.00), *RERG* (OR = 2.08, 95% CI: 1.56–2.77, *P* = 0.00), *ADAMTS9* (OR = 1.85, 95% CI: 1.37–2.49, *P* = 0.00) and *IRF4* (OR = 16.96, 95% CI: 5.15–55.84, *P* = 0.00) were still significantly associated with CRC. However, no statistically significant associations were found between the methylation status of *FOXE-1* (*P* = 0.06) or *RARB2* (*P* = 0.78) and CRC.

The results of stratification analysis by age suggested that hypermethylation of *FOXE-1* and *AOX-1* were associated with risk of CRC only in the older group (aged 60 yrs and older). Hypermethylation of *ADAMTS9* and *RERG* were significantly associated with risk of CRC in both the young and old group, with stronger associations in the old group (Table 3).

In another stratification analysis by family history of cancer, *AOX-1* hypermethylation was associated with risk of CRC regardless of the presence of a family history of cancer. Hypermethylation of *ADAMTS9* and *RERG* were associated with risk of CRC only without a family history of cancer (Table 4).

### Association between MCSM methylation status and CRC

These candidate gene biomarkers were integrated into the multiple CpG site methylation (MCSM) panel. High-level MCSM (MCSM-H) was defined when 2 or more of the 5 candidate genes (*AOX-1*, *RERG*, *ADAMTS9*, *IRF4* or *FOXE-1*) were methylated; low-level MCSM (MCSM-L) was defined as 1 of the 5 candidate genes being methylated; and non-MCSM was defined as none of the 5 candidate genes being methylated.

Multivariate logistic regression analysis suggested that there was a statistically significant difference in the distribution of cases and controls in the non-MCSM and MCSM groups (OR = 1.50, 95% CI: 1.11–2.03, *P* = 0.01). Moreover, after dividing MSCM into two groups, MCSM-L and MCSM-H, a stronger association between MCSM-H (compared with non-MCSM) and CRC (OR = 1.79, 95% CI: 1.28–2.52, *P* = 0.00) was observed (Table 2).

Compared to the non-MCSM group, MCSM was associated with risk of CRC in the older group (aged older than 60 yrs) and without family history of cancer in a stratification analysis (*P* = 0.000 and *P* = 0.002, respectively; Tables 3 and 4). MCSM-L was not associated with risk of CRC without family history of cancer (*P* = 0.225; Table 4).

The population attributable risk percentage (PAR%) of MCSM on the risk of CRC was 26.1%.

### Interaction between the methylation of individual genes, MCSM and environmental factors on the risk of CRC

Antagonistic effects of *ADAMTS9* hypermethylation and consumption of stewed fish with brown sauce (≥1 times/week) on the risk of CRC were observed (OR = 0.50, 95% CI: 0.25–0.99, *P* = 0.046; Supplementary Data Table S10) compared with unmethylated *ADAMTS9* and consumption of stewed fish with brown sauce (<1 times/week).

Significant synergistic effects between *AOX-1* hypermethylation and intake of cereals (≥100 g/week) and age (older than 60 yrs) on risk of CRC were observed (OR = 1.82, 95% CI: 1.01–3.26, *P* = 0.045 and OR = 3.21, 95% CI: 1.83–5.64, *P* = 0.00, respectively; Table 5 and Supplementary Data Table S5).

For *RARB2* hypermethylation, antagonistic interactions with pungent food (≥4 times/week), or intake of food overnight (≥3 times/week) were observed (OR = 0.52, 95% CI: 0.28–0.99, *P* = 0.047 and OR = 0.48, 95% CI: 0.25–0.93, *P* = 0.03, respectively; Supplementary Data Table S13 and S15).

Additionally, a synergistic effect between *RERG* hypermethylation and age (older than 60 yrs) on risk of CRC was observed (OR = 2.65, 95% CI: 2.07–3.38, *P* = 0.00; Table 5).

Furthermore, MCSM showed a statistically significant synergistic interaction with age (older than 60 yrs; OR = 2.99, 95% CI: 21.60–5.59, *P* = 0.00; Table 5).

The models assessing the association between the six genes, MCSM and environmental factors on CRC were presented in Tables 5 and Supplementary Data Table S5–S16.

### Association between the methylation of individual genes, MCSM and CRC prognosis

At the end of the follow-up time (109 months), 113/256 (44.3%) of the patients were confirmed to be alive and 113/256 (44.3%) deaths had occurred. The mean overall survival (OS) of CRC patients was 59.2 months. Multiple Cox regression analysis suggested that Dukes staging, preoperative CA19-9 level and intestinal anastomosis were significantly associated with prognosis of CRC (Supplementary Data Table S3). However, no statistically significant association between the methylation status of the six genes, MCSM and the prognosis of CRC were observed either before or after adjusting for Dukes staging, preoperative carbohydrate antigen 19-9 (CA19-9) level and intestinal anastomosis (Supplementary Data Table S4).

## Discussion

DNA methylation occurs in genomic regions rich in cytosine and guanosine (CG) dinucleotides, called CpG islands. This is known to be a well-characterized event in tumor biology and has been extensively documented in CRC tissues^24^,^34^,^35^. Aberrant DNA hypermethylation of specific genes may result in abnormal expression in normal cells, especially at tumor suppressor genes, whose hypermethylation is related to the down-regulation of gene expression, leading to the proliferation and differentiation of tumor cells. Therefore, we proposed that DNA hypermethylation in leukocytes in the peripheral blood may be used as a biomarker of CRC, as described previously^36^. Ally *et al*. studied the relationship between estrogen receptor gene methylation in leukocytes and normal colonic tissue DNA in subjects with and without colorectal neoplasia, and found that methylation in leukocytes was 60% lower than that in normal colonic tissue (*P* < 0.001)^37^. To explore the association between methylation status in leukocyte DNA and CRC, Gao *et al*. found 31 CpG sites that were significantly related to CRC risk, especially at two sites in the *DSP* gene in male smokers^38^. The candidate biomarkers involved in our studies have been shown to be involved in multiple molecular events associated with tumorigenesis, among which *IRF4* hypermethylation associated with the highest risk of CRC (OR = 16.96), while the lowest risk was seen for *FOXE-1* hypermethylation (OR = 1.35). Next, we defined a gene panel to assess methylation called MCSM, which included 5 candidate genes, and we found that people with MCSM hypermethylation were 1.54 times more susceptible to CRC compared with non-MCSM hypermethylation (*P* = 0.004). The high PAR% (26.1%) of MCSM hypermethylation suggests that these methylated genes play a key role in CRC risk.

Dietary factors are commonly recognized as modifiable factors that profoundly influence cancer and tumor behavior^39^, accounting for 70% the risk of CRC. To date, many researchers have reported that dietary factors and lifestyle not only play a crucial role in tumorigenesis, but are also associated with inducing epigenetic changes^40^. Therefore, we explored the interaction of dietary factors exposure and peripheral blood DNA methylation level on susceptibility to CRC.

Although the antagonistic interactions between increased vegetable intake (≥100 g/day) and individual genes, MCSM showed no statistical significance, the combinations showed a protective tendency on risk of CRC. Folic acid, rich in vegetables, plays an important role in the provision of methyl moieties that are used to synthesize S-adenosyl methionine, which is the universal methyl donor for DNA methylation^41^. Inadequate folate availability during cell division can result in the compromised production of thymidine, such that uracil may be substituted in the DNA sequence, which may trigger repair attempts that increases the frequency of chromosomal breaks^42^. Moreover, methionine, as the substrate for SAM and human essential amino acids, is also critical for maintaining the flux of methyl groups for rementhylation^41^. Therefore, low dietary intake levels with long-term methionine and folate deficiency could result in methionine cycle disorder, which can be confused with DNA methylation, leading normally non-methylated DNA situation to be aberrantly hypermethylated^43^. In addition, we observed antagonistic effects between *ADAMTS9* hypermethylation and consumption of stewed fish with brown sauce, likely due to the abundant methionine found in fish.

Carcinogenic N-nitroso compounds, which could induce mutations by alkylating DNA and thus activating oncogenes, can be found in food left overnight and are endogenously formed after ingesting red meat in the intestines with the help of the colonic flora^44^,^45^. *In vivo* studies in rat livers showed alterations of DNA methylation patterns by N-Nitrosodimethylamine (NDMA) and it was found that 6.6% of O^6^-methyl-guanine-O^6^ meG (O^6^) position conferred a high mutagenic and carcinogenic susceptibility^46^, which suggested the possibility that the interaction between eating a lot of food left overnight and *RARB2* hypermethylation observed in our study was associated with the risk of CRC.

Some of the effects of the qualitative and quantitative aspects of fat intake have imputed to be a modification of the transcription of key genes involved in pathways related to lipid and glucose metabolism^47^. Abundant amounts of *AOX1* have been observed in adipose tissue and were proposed to play a critical role in adipogenesis and lipid metabolism by modulating peroxisome proliferator-activated receptor-α^48^,^49^, which may provide a basis for explaining why fat intake showed an antagonistic interaction with *AOX-1* hypermethylation on the risk of CRC. However, the molecular mechanisms relating dietary factors and DNA hypermethylation to effects on tumor carcinogenic process are very complex, and further studies are needed.

No significant relationships were observed between the methylation status of individual genes, MCSM and prognosis of CRC. It has been shown that aberrant hypermethylation of *RARB2* in tumor tissue was associated with a shortened OS in 73 CRC tissues^50^. A meta-analysis showed that CIMP was independently associated with significantly worse prognosis in CRC patients^51^, suggesting that the mechanism by which CIMP from CRC tissues and MCSM from peripheral blood cells influences CRC prognosis may be different.

Although we did not validate the results of methylation-sensitive high-resolution melting analysis (MS-HRM) with another technique in this study, we did test *DAPK-1* and *MLH-1* methylation status using MS-HRM, validated with pyrosequecing in our previous study. For *DAPK-1*, the sensitivity was 89.74% and the specificity was 72.34%. For *MLH-1*, the sensitivity was 100% and the specificity was 78.69%. This suggests that using MS-HRM to test methylation status was stable and the results were credible.

There were several limitations in our study. First, recall bias may be inevitable for the collection of information on environmental factors, although we did our best to minimize this bias. Second, the collection of information about dietary intake without detailed amounts may limit our power to detect gene-dietary interactions more precisely. Until now, it has not been clear how DNA methylation produces effects on gene expression and to what degree specific DNA methylation can lead to changes in gene expression, eventually resulting in changes in individual susceptibility to diseases.

In summary, our study suggests that both individual gene methylation of *IRF4*, *FOXE-1*, *AOX-1*, *ADAMTS9* and *RERG* as well as MCSM in peripheral blood leukocytes are associated with increased risk of CRC but are not associated with the prognosis of CRC. Hypermethylation variability detected in leukocytes may interact with dietary factors and affect susceptibility to CRC. Additional large-scale studies will be required to validate these observations. Screening additional candidate genes using a more systematic method and synthesizing additional experimental results for further study of MCSM biomarkers in peripheral blood from CRC cases are needed.

## Materials and Methods

### Study population

We carried out this study after obtaining informed written consent from study subjects and approval from the Human Research and Ethics Committee of Harbin Medical University. All experiments including all relevant details were performed in accordance with relevant guidelines and regulations. We identified 421 CRC patients who underwent surgery at the Cancer Hospital (from June 1, 2004 to May 15, 2005, and May 15, 2007 to January 1, 2008) and the second Affiliated Hospital of Harbin Medical University (from October 15, 2010 to June 15, 2011). All patients were diagnosed based on pathology. Patients with neuroendocrine carcinomas, malignant melanomas, non-Hodgkin’s lymphoma, gastrointestinal stromal tumors, metastatic CRC, and Lynch syndrome CRC were excluded. The 506 control subjects were selected from the Departments of Orthopedics and Ophthalmology at the Second Affiliated Hospital of Harbin Medical University during the same period. Patients with gastrointestinal disease or CRC history were excluded. Samples of peripheral blood (5 ml) were collected and stored at −80 °C immediately. Each participant was interviewed in person by a well-trained interviewer using a structured questionnaire according to the dietary habits in north China and a nutrition survey from the Chinese National Survey Data Archive with information of on demographic characteristics (age, gender, height and weight, education and occupation), lifestyle factors (use of tobacco, alcohol and smoking) and family history of cancer as well as dietary status during the past 12 months.

Postoperatively, we carried out a cohort study within the 256 patients from the Cancer Hospital. The patients were followed at 3–6-month intervals for the first year after resection, and annually thereafter. Clinical information about Dukes stage, chemotherapy, histological and pathological types, the level of serum carcinoembryonic antigen (CEA) and CA19-9 before surgery were collected from medical records. OS that was defined as the time from surgery to the patient’s death or the last follow-up visit was used as a measure of prognosis. The date of the last follow-up was March 15, 2014 (the 109^th^ month). The date and cause of death of CRC patients were validated. The rate of loss to follow-up was 11.4% (29/256).

### Genomic DNA extraction and sodium bisulfite modification

Genomic DNA was successfully extracted from the peripheral blood samples of 421 CRC patients and 506 controls using the QIAamp DNA Blood Mini kit (Qiagen, Hilden, Germany) and was stored at −80 °C. DNA quantity was measured using the Nanodrop 2000 Spectrophotometer (Thermo Scientific).

Bisulfite treatment was used to transform unmethylated cytosine nucleotides into thymidine without changing methylated cytosines. Genomic DNA was chemically modified with a sodium bisulfite modification kit (EpiTect Fast DNA Bisulfite Kit, Qiagen, Hilden, Germany), according to the manufacturer’s instructions. The bisulfate- modified DNA was stored at −20 °C until use.

### Analysis of the methylation status of candidate genes

Methylation-sensitive high-resolution melting analysis (MS-HRM) was performed on a LightCycler480 machine (Roche Applied Science, Mannheim, Germany) as previously described^36^. The primers are listed in Table 6. HRM was performed in a 10 μl volume system consisting of 1X LightCycler480 High Resolution Melting Master Mix (Roche), 200 nmol/l of each primer, and 5 ng of a sodium bisulfite-modified DNA template. The final MgCl_2_ concentration was adjusted to 3 mmol/l. The PCR amplification protocol consisted of denaturation for 10 min at 95 °C for 1 cycle, denaturation for 10 s at 95 °C, annealing for 30 s with a touchdown of each primer annealing temperature and extension for 30 s at 72 °C for 45 cycles. The HRM melting protocol then consisted of 95 °C for 1 min, 40 °C for 1 min, 74 °C for 5 s and continuous acquisition to 90 °C at 25 acquisitions per 1 °C (LightCycler480, Roche, Mannheim, Germany).

**Figure 1:**
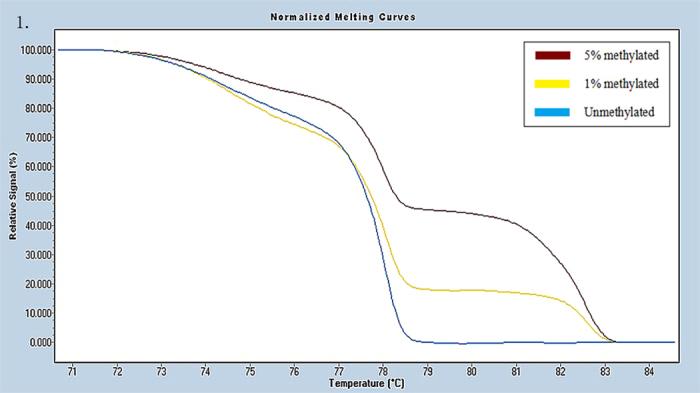
Profile of fluorescence obtained at the melting temperature for serial dilutions of methylated DNA (5%, 1%, and 0%) in ADAMTS9 gene.

**Figure 2:**
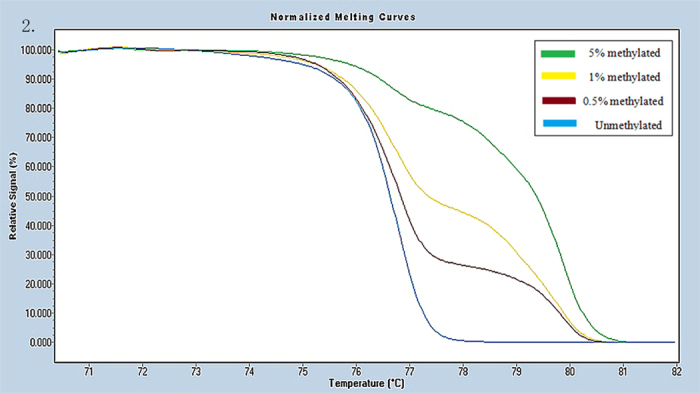
Profile of fluorescence obtained at the melting temperature for serial dilutions of methylated DNA (5%, 1%, 0.5% and 0%) in AOX-1 gene.

**Figure 3:**
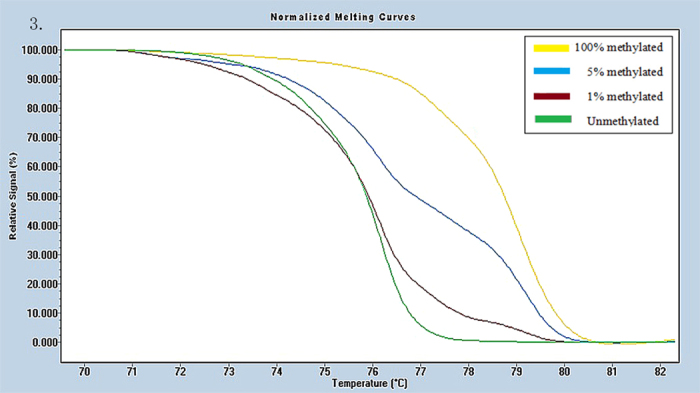
Profile of fluorescence obtained at the melting temperature for serial dilutions of methylated DNA (100%, 5%, 1%, and 0%) in FOXE-1 gene.

**Figure 4:**
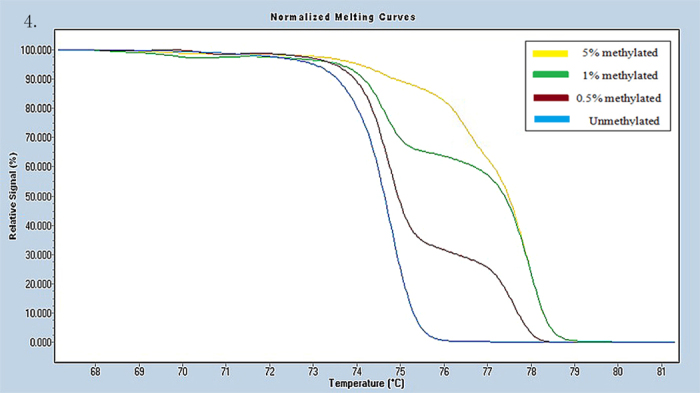
Profile of fluorescence obtained at the melting temperature for serial dilutions of methylated DNA (5%, 1%, 0.5% and 0%) in IRF4 gene.

**Figure 5:**
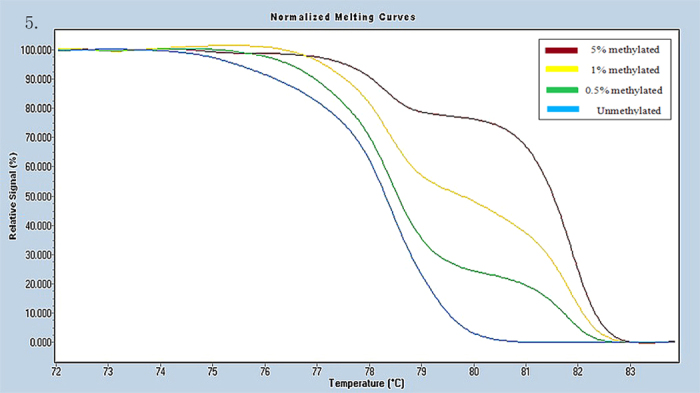
Profile of fluorescence obtained at the melting temperature for serial dilutions of methylated DNA (5%, 1%, 0.5% and 0%) in RARB2 gene.

**Figure 6:**
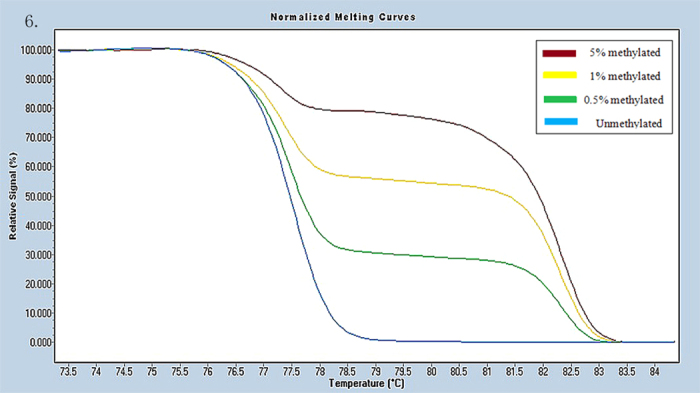
Profile of fluorescence obtained at the melting temperature for serial dilutions of methylated DNA (5%, 1%, 0.5% and 0%) in RERG gene.

Universal methylated (100% methylated) and unmethylated (0% methylated) human whole genomic DNA samples (Zymo Research) were used as a calibrator and positive control, respectively. DNA-free distilled water was used as a negative control. A series of methylated DNA, including 100%, 5%, 1%, 0.5% and 0% on a background of universal unmethylated DNA were constructed as standard curves by serially diluting the methylated control DNA into the unmethylated control according to mass concentration (Figs 1, 2, 3, 4, 5, 6).

HRM data were analyzed using Gene Scanning Software (version 2.0). Data processing included normalization and temperature shifting using a LightCycler480. The methylation status of the candidate genes was determined by comparing the curves of each sample to the series of standard dilutions in the gene scanning module by two independent observers. Disagreements were settled by consensus or a third opinion.

### Statistical analyses

Continuous variables such as age were analyzed by two-sample t*-*tests. The statistical significance of the association between categorical variables was assessed with chi-square (*χ*^*2*^) tests. We determined 0% as the cutoff value of different methylation status for comparisons between CRC patients and controls. Univariate and multivariate logistic regression analyses were used to assess if relationships existed between the methylation status of *AOX-1*, *ADAMTS9*, *FOXE-1*, *IRF4*, *RARB2* and *RERG*, as well as MCSM and CRC. Multiple imputation was applied to generate possible values for missing values of dietary factors by creating several “complete” sets of data. We selected the pooled output of each “complete” dataset analysis for subsequent analyses. Multivariate logistic regression analysis and crossover analysis were performed to evaluate the role of candidate genes and MCSM methylation status in interaction with and in combination with environmental factors on the risk of CRC with 4 types of ORs: one comparing exposed and unexposed subjects to the environmental risk factor (OR_e_); one for the association between gene methylation and risk of CRC (OR_g_); one for combining the effects of both gene hypermethylation and environmental factos (OR_eg_); and one for the interaction between DNA methylation and environmental factors (OR_i_ = OR_eg_/OR_e_OR_g_). The reference groups consisted of subjects without exposure to the environmental factors or gene methylation. OS was estimated using the life table method. Survival curves were constructed using the Kaplan-Meier method. The Cox regression model was performed to compute the hazard ratio (HR) and 95% CI for the potential prognostic factors. The population attributable risk percentage (PAR %) was calculated by P_exposed_ (OR − 1)/[1 + P_exposed_(OR − 1)]. All statistical analyses were performed using SAS 9.2 software (SAS Institute, Cary, NC, USA). The results were considered significantly different if statistical tests produced a two-tailed *P*-value of < 0.05.

## Additional Information

**How to cite this article**: Luo, X. *et al*. Methylation of a panel of genes in peripheral blood leukocytes is associated with colorectal cancer. *Sci. Rep.* **6**, 29922; doi: 10.1038/srep29922 (2016).

## Supplementary Material

Supplementary Information

## Figures and Tables

**Table 1:** Characteristics of colorectal cancer patients and controls.

Variable	Cases (%) (n = 421)	Controls (%) (n = 506)	*P* values
Age (mean ± SD)	59.46 ± 11.41	56.62 ± 10.97	0.000
<50	79 (18.8)	133 (26.3)	0.001
50–59	129 (30.6)	175 (34.6)	
60–69	126 (29.9)	132 (26.1)	
≥70	87 (20.7)	66 (13.0)	
Gender			
Male	258 (61.3)	279 (55.1)	0.059
Female	163 (38.7)	227 (44.9)	
BMI (kg/m^2^)			
≤18.50	32 (7.7)	26 (5.2)	0.003
18.5–23.00	167 (40.0)	156 (31.4)	
≥23.00	219 (52.4)	315 (63.4)	
Education			
Primary and below	114 (29.2)	157 (32.2)	0.576
Junior high school	115 (29.4)	148 (30.4)	
Senior middle school	84 (21.5)	100 (20.5)	
College and above	78 (19.9)	82 (16.8)	
Occupation			
Mental worker	170 (43.9)	246 (52.9)	0.021
Manual worker	116 (30.0)	127 (27.3)	
Combined	101 (26.1)	92 (19.8)	
Family history of cancer			
No	312 (78.4)	307 (86.5)	0.004
Yes	86 (21.6)	48 (13.5)	
Tumor location			
Colon	117 (33.5)	—	—
Rectum	232 (66.5)	—	

**Table 2:** Associations between methylation of individual genes, MCSM and the risk of CRC.

Gene		Case (%)	Control (%)	OR	95% CI	*P*value	OR^a^	95% CI	*P*value
IRF4	Unmethylation	381 (90.5)	495 (99.4)	1.00			1.00		
	Methylation	40 (9.5)	3 (0.6)	17.32	5.32–56.42	0.000	16.96	5.15–55.84	0.00
FOXE-1	Unmethylation	301 (71.5)	388 (78.5)	1.00			1.00		
	Methylation	120 (28.5)	106 (21.5)	1.46	1.08–1.97	0.014	1.35	0.99–1.85	0.06
AOX-1	Unmethylation	214 (51.2)	312 (63.5)	1.00			1.00		
	Methylation	204 (48.8)	179 (36.5)	1.66	1.27–2.17	0.000	1.72	1.30–2.27	0.00
ADAMTS9	Unmethylation	268 (64.0)	387 (77.4)	1.00			1.00		
	Methylation	151 (36.0)	113 (22.6)	1.93	1.45–2.58	0.000	1.85	1.37–2.49	0.00
RERG	Unmethylation	240 (57.3)	370 (74.1)	1.00			1.00		
	Methylation	179 (42.7)	129 (25.9)	2.14	1.62–2.83	0.000	2.08	1.56–2.77	0.00
RARB2	Unmethylation	314 (74.8)	377 (75.2)	1.00			1.00		
Methylation	106 (25.2)	124 (24.8)	1.03	0.76–1.39	0.865	0.96	0.70–1.30	0.78	
MCSM	Non-MCSM	106 (25.5)	167 (34.5)	1.00			1.00		
	MCSM-L	125 (30.1)	161 (33.3)	1.22	0.87–1.71	0.242	1.23	0.87–1.75	0.24
	MCSM-H	184 (44.3)	156 (32.2)	1.86	1.34–2.57	0.000	1.79	1.28–2.52	0.00
	MCSM	309 (74.5)	317 (65.5)	1.54	1.15–2.05	0.004	1.50	1.11–2.03	0.01

^a^Adjusted for age, BMI, occupation and family history of cancer.

**Table 3:** Association between methylation of genes and risk of CRC by age.

Gene	<60			≥60		
	OR^a^	95% CI	*P*value	OR^a^	95% CI	*P*value
IRF4	9.618	1.187–77.931	0.034	−	−	−
FOXE-1	0.977	0.571–1.670	0.932	1.700	1.023–2.824	0.040
AOX-1	1.540	0.972–2.440	0.066	3.791	2.301–6.246	0.000
ADAMTS9	1.803	1.086–2.992	0.023	2.025	1.244–3.296	0.005
RERG	2.036	1.236–3.355	0.005	4.821	2.824–8.231	0.000
RARB2	0.666	0.405–1.096	0.110	0.921	0.556–1.526	0.750
MSCM-L	1.104	0.661–1.844	0.706	1.938	1.027–3.655	0.041
MSCM-H	1.527	0.886–2.634	0.128	5.128	2.744–9.584	0.000
MSCM	1.276	0.814–2.002	0.288	3.334	1.907–5.830	0.000

^a^Adjusted for BMI, occupation and family history of cancer.

^a^All the ORs were calculated by selecting unmethylation as a reference group.

**Table 4:** Association between methylation of genes and risk of CRC by family history of cancer.

Gene	No			Yes		
	OR^a^	95% CI	*P*value	OR^a^	95% CI	*P*value
IRF4	25.143	3.350–188.705	0.002	—	—	—
FOXE-1	1.425	0.954–2.130	0.084	0.845	0.345–2.066	0.712
AOX-1	2.340	1.622–3.374	0.000	2.467	1.012–6.010	0.047
ADAMTS9	1.871	1.277–2.740	0.001	1.868	0.773–4.517	0.165
RERG	3.269	2.195–4.869	0.000	2.215	0.905–5.423	0.082
RARB2	0.757	0.512–1.121	0.165	0.932	0.412–2.107	0.866
MSCM-L	1.311	0.846–2.032	0.225	1.288	0.518–3.201	0.585
MSCM-H	2.606	1.669–4.069	0.000	2.661	0.984–7.194	0.054
MSCM	1.839	1.253–2.701	0.002	1.793	0.803–4.002	0.154

^a^Adjusted for age, BMI and occupation.

^a^All the ORs were calculated by selecting unmethylation as a reference group.

**Table 5:** Effects of combination and interaction between age and methylation of genes, MCSM on the risk of CRC.

	Age			
	<60	≥60	Interaction	
	OR_eg_^a^ (95% CI)		OR_i_^a^ (95% CI)	*P*
*FOXE-1*				
Unmethylation	1.00	1.28 (1.12–1.48)		
Methylation	1.11 (0.91–1.35)	2.23 (1.85–2.70)	1.57 (0.84–2.94)	0.16
*IRF4*				
Unmethylation	1.00	1.40 (1.24–1.59)		
Methylation	10.66 (5.43–20.95)	41.76 (17.00–102.59)	2.80 (0.23–34.61)	0.42
*ADAMTS9*				
Unmethylation	1.00	1.25 (1.08–1.45)		
Methylation	1.54 (1.28–1.86)	2.87 (2.39–3.45)	1.49 (0.82–2.72)	0.19
*AOX1*				
Unmethylation	1.00	0.83 (0.70–0.98)		
Methylation	1.03 (0.87–1.22)	2.75 (2.32–3.25)	3.21 (1.83–5.64)	0.00
*RERG*				
Unmethylation	1.00	1.02 (0.88–1.19)		
Methylation	1.36 (1.14–1.62)	3.68 (3.06–4.43)	2.65 (2.07–3.38)	0.00
*RARB2*				
Unmethylation	1.00	1.51 (1.31–1.74)		
Methylation	0.99 (0.82–1.19)	1.46 (1.21–1.78)	1.02 (0.79–1.32)	0.87
MCSM				
Unmethylation	1.00	0.66 (0.52–0.83)		
Methylation	1.01 (0.85–1.19)	1.98 (1.66–2.35)	2.99 (1.60–5.59)	0.00

^a^Adjusted for BMI, occupation and family history of cancer.

**Table 6:** Primers for MS-HRM.

Gene	Primer sequences (5′-3′)	Location	Number of CpG-sites/Length of amplified fragment	Annealing temperature (°C)
AOX-1	F: GAACGTTGGATTTTAATTAAGGTTTTTR: CTAAAAAATAACGAACACCTAAAACC	2q33	8/124	64–56
ADAMTS9	F: ATTTGGTCGAGATGGGGAGTTR: CTACGAAATACCCCTACCCAA	3p14	16/151	62–56
FOXE-1	F: GATTTTTTAGTGAATGGTTAGGGR: CTACTATCCCTAAACCGAAACTT	9q22	6/96	68–58
IRF4	F: CGTTGTAGTTTAGTGATTGATTGR: GCTTCGAAAACTATCACTAAAAC	6p25.3	8/111	62–56
RARB2	F: CGAGTTGTTTGAGGATTGGGATGTR: AATACGTTCCGAATCCTACCCC	3p24	7/89	66–56
RERG	F: CGGTTTTGGTCGGGTTTAGTTR: CGCAAAAACAAATACCAATAACC	12p12	11/107	64–56
