# Supplementary material for: Methylation of a panel of genes in peripheral blood leukocytes is associated with colorectal cancer
Source: Sci Rep. 2016 Jul 25;6:29922. doi: 10.1038/srep29922 (PMC4958953; doi:10.1038/srep29922)
Supplement: Supplementary Information [file srep29922-s1.pdf]

Methylation of a panel of genes in peripheral blood leukocytes is associated with colorectal cancer

Xiang Luo[1], Rong Huang[1], Hongru Sun[1], Yupeng Liu[1], Haoran Bi[1], Jing Li[1], Hongyuan Yu[1], Jiamei Sun[1], Shangqun Lin[2], Binbin Cui[2], Yashuang Zhao[1]

Supplementary data

Table S1 Associations between exposure factors and CRC risk

Factor		Case (%)	Control (%)	OR	95%CI	*P*	OR[a]	95%CI	*P*	OR[b]	95%CI	*P*
Flour and rice	<100g /Day	10 (2.4)	14 (2.8)	1.00								
	≥100g /Day	406 (97.6)	491 (97.2)	1.15	0.50-2.61	0.74	1.02	0.52-2.80	0.67	1.02	0.52-2.80	0.67
Cereals	<100g /Week	293 (70.3)	298 (59.4)	1.00								
	≥100g /Week	124 (29.7)	204 (40.6)	0.62	0.47-0.81	0.00	0.53	0.40-0.71	0.00	0.53	0.40-0.71	0.00
Vegetables	<100g /Day	43 (10.5)	31 (6.2)	1.00								
	≥100g /Day	366 (89.5)	473 (93.8)	0.56	0.36-0.90	0.02	0.63	0.38-1.03	0.07	0.63	0.38-1.04	0.07
Fruits	<3 Times/Week	189 (45.4)	232 (46.2)	1.00								
	≥3 Times/Week	227 (54.6)	270 (53.8)	1.03	0.93-1.15	0.52	0.96	0.84-1.09	0.48	0.96	0.84-1.09	0.48
Fat	No	189 (46.6)	283 (56.8)	1.00								
	Yes	217 (53.4)	215 (43.2)	1.53	1.18-1.99	0.00	1.58	1.20-2.08	0.00	1.56	1.19-2.05	0.00
Beef and mutton	<250g /Week	353 (85.9)	391 (80.3)	1.00								
	≥250g /Week	58 (14.1)	96 (19.7)	0.69	0.48-0.98	0.04	0.74	0.51-1.07	0.11	0.74	0.51-1.07	0.11
Pork	<250g /Week	202 (49.8)	281 (57.9)	1.00								
	≥250g /Week	204 (50.2)	204 (42.1)	1.41	1.08-1.83	0.01	1.49	1.14-1.96	0.00	1.49	1.13-1.95	0.00
	<250g /Week	352 (88.7)	411 (87.4)	1.00								

Poultry	≥250g /Week	45 (11.3)	59 (12.6)	0.88	0.58-1.34	0.56	0.94	0.61-1.43	0.76	0.94	0.61-1.43	0.76
Fish	<2 Times/Month	182 (44.1)	244 (48.3)	1.00								
	≥2 Times/Month	231 (55.9)	261 (51.7)	1.17	0.90-1.52	0.24	1.11	0.84-1.45	0.47	1.11	0.84-1.45	0.47
Seafood	<1 Times/Month	310 (82.4)	405 (86.5)	1.00								
	≥1 Times/Month	66 (17.6)	63 (13.5)	1.40	0.97-2.02	0.07	1.47	0.997-2.17	0.052	1.47	0.99-2.17	0.05
Stewed fish with brown sauce	<1 Times/Week	282 (68.3)	382 (77.6)	1.00								
	≥1 Times/Week	131 (31.7)	110 (22.4)	1.59	1.18-2.13	0.00	1.65	1.21-2.24	0.00	1.65	1.21-2.24	0.00
Milk	<3 Times/Week	268 (64.9)	226 (58.2)	1.00								
	≥3 Times/Week	145 (35.1)	162 (41.8)	0.80	0.59-1.09	0.15	0.66	0.47-0.93	0.02	0.66	0.47-0.93	0.02
Egg	No	44 (10.7)	48 (9.6)	1.00								
	Yes	369 (89.3)	454 (90.4)	0.89	0.58-1.36	0.58	0.80	0.52-1.25	0.34	0.80	0.52-1.25	0.34
Coffee	No	399 (96.8)	478 (94.7)	1.00								
	Yes	13 (3.2)	27 (5.3)	0.57	0.29-1.12	0.10	0.51	0.25-1.05	0.07	0.51	0.25-1.05	0.07
Tea	No	297 (71.7)	378 (74.9)	1.00								
	Yes	117 (28.3)	127 (25.1)	1.19	0.89-1.59	0.25	1.14	0.83-1.56	0.42	1.13	0.83-1.54	0.45
Sodas	No	395 (96.8)	484 (95.8)	1.00								
	Yes	13 (3.2)	21 (4.2)	1.31	0.65-2.64	0.45	1.20	0.58-2.47	0.63	1.20	0.58-2.47	0.63
Fried food	<1 Times/Month	293 (70.6)	403 (80.0)	1.00								
	≥1 Times/Month	122 (29.4)	101 (20.0)	1.68	1.24-2.28	0.00	1.68	1.23-2.30	0.00	1.68	1.23-2.90	0.00

Smoked food	<1 Times/Month	372 (89.9)	434 (87.9)	1.00								
	≥1 Times/Month	42 (10.1)	60 (12.1)	0.83	0.55-1.26	0.38	0.91	0.59-1.42	0.68	0.91	0.59-1.41	0.68
Sausages	<1 Times/Month	173 (73.9)	400 (89.1)	1.00								
	≥1 Times/Month	61 (26.1)	49 (10.9)	2.55	1.69-3.85	0.00	3.31	2.18-5.02	0.00	3.24	2.15-4.90	0.00
Pungent food	<4 Times/Week	235 (56.6)	242 (48.2)	1.00								
	≥4 Times/Week	180 (43.4)	260 (51.8)	0.72	0.55-0.94	0.02	0.76	0.57-0.99	0.045	0.76	0.57-0.99	0.05
Garlic	<4 Times/Week	242 (58.3)	282 (55.8)	1.00								
	≥4 Times/Week	173 (41.7)	223 (44.2)	0.92	0.70-1.19	0.51	0.95	0.72-1.24	0.69	0.94	0.72-1.24	0.68
Sauerkraut	<3 Times/Week	180 (43.5)	151 (30.0)	1.00								
	≥3 Times/Week	234 (56.5)	352 (70.0)	0.56	0.43-0.73	0.00	0.60	0.45-0.80	0.00	0.60	0.45-0.80	0.00
Canned fruit	No	364 (91.2)	418 (92.9)	1.00								
	Yes	35 (8.8)	32 (7.1)	1.30	0.78-2.15	0.31	1.22	0.73-2.06	0.45	1.22	0.83-2.06	0.45
Canned meat	No	376 (94.5)	423 (93.8)	1.00								
	Yes	22 (5.5)	28 (6.2)	0.86	0.48-1.55	0.62	0.91	0.50-1.64	0.75	0.99	0.66-1.48	0.96
Overnight food	<3 Times/Week	231 (55.7)	332 (66.4)	1.00								
	≥3 Times/Week	184 (44.3)	168 (33.6)	1.57	1.20-2.05	0.00	1.64	1.24-2.17	0.00	1.64	1.24-2.17	0.00
Smoking	No	235 (56.8)	290 (57.4)	1.00								
	Yes	179 (43.2)	215 (42.6)	1.04	0.80-1.35	0.77	0.98	0.75-1.29	0.89	0.98	0.75-1.29	0.89
	No	134 (57.5)	254 (59.1)	1.00								

Drinking	Yes	99 (42.5)	176 (40.9)	0.98	0.65-1.48	0.92	0.96	0.57-1.59	0.85	0.96	0.62-1.46	0.82

[a] Adjusted for age, BMI, occupation, family history of cancer and smoking

[b] Adjusted for age, BMI, occupation, family history of cancer

Table S2 Clinicopathologic characteristics of CRC patients

Clinicopathologic characteristics	Patients (n=256) (%)
Age	
＜50	52 (20.4)
50-59	80 (31.4)
60-69	72 (28.2)
≥70	51 (20.0)
Gender	
Male	145 (56.9)
Female	110 (43.1)
Dukes stage	
A	30 (11.8)
B	111 (43.7)
C	95 (37.4)
D	18 (7.1)
Pathological grade	
Low	40 (15.7)
Medium	201 (78.8)
High	2 (0.8)
Unknown	12 (4.7)
Tumor location	
Colon	81 (32.1)
Rectum	171 (67.9)
Metastasis status	
M0	114 (44.7)

M1	141 (55.3)
Multiple polyps	
No	167 (70.2)
Yes	71 (29.8)
CEA (carcino-embryonic antigen) level before operation (ng/ml)	
<5	111 (43.5)
≥5	144 (56.5)
CA19-9(carbohydrate antigen 19-9) level before operation (μg/ml)	
<37	183 (73.2)
≥37	67 (26.8)
Histologic type	
Adenocarcinoma	245 (99.2)
Other types	2 (0.8)
Pathogenic type	
Protrude type	159 (64.4)
Ulcerative or infiltrating type	86 (34.8)
Other type	2 (0.8)
Intestinal anastomosis	
No	63 (26.6)
Yes	174 (73.4)

Table S3 Cox regression analysis with association between clinicopathologic factors and CRC prognosis

	B	SE	Wald	df	*P* value	HR	95% CI
Dukes (A)			18.038	3	0.000		
Dukes (B)	0.749	0.509	2.166	1	0.141	2.116	0.780-5.740
Dukes (C)	0.546	0.947	0.333	1	0.564	1.727	0.270-11.052
Dukes (D)	1.937	1.024	3.576	1	0.059	6.935	0.932-51.619
Multiple polyps	0.161	0.249	0.417	1	0.518	1.174	0.721-1.912
Metastasis status	-0.912	0.792	1.328	1	0.249	0.402	0.085-1.895
Pathological grade (Low)			3.664	3	0.300		
Pathological grade (Medium)	-0.418	0.289	2.092	1	0.148	0.658	0.373-1.160
Pathological grade (High)	-0.644	0.774	0.693	1	0.405	0.525	0.115-2.393
Pathological grade (Unkown)	-0.966	0.595	2.634	1	0.105	0.381	0.118-1.222
Preoperative CEA level	0.088	0.271	0.106	1	0.745	1.092	0.642-1.858
Pathogenic type (Protrude type)			0.354	2	0.838		
Pathogenic type (Ulcerative or infiltrating type)	-0.008	0.295	0.001	1	0.978	0.992	0.556-1.768
Pathogenic type (Other type)	0.318	0.545	0.340	1	0.560	1.374	0.472-4.000
Preoperative CA19-9 level	1.459	0.261	31.179	1	0.000	4.300	2.577-7.176
Intestinal anastomosis	0.974	0.249	15.263	1	0.000	2.648	1.625-4.315
Intraoperative chemotherapy	-0.175	0.237	0.548	1	0.459	0.839	0.528-1.335
Postoperative chemotherapy	0.221	0.240	0.852	1	0.356	1.248	0.780-1.997

Table S4 Associations between methylation of individual genes, MCSM and prognosis of CRC

Gene		Patients (n=256) (%)	3-year Survival (%)	5-year Survival (%)	OS (months)	HR (95% CI)	*P* value	HR[a] (95% CI)	*P* value
IRF4	Unmethylation	228 (89.4)	67	58	73.63±2.70	1.00		1.00	
	Methylation	27 (10.6)	67	63	70.25±7.89	0.93 (0.50-1.73)	0.82	0.78 (0.41-1.49)	0.45
FOXE-1	Unmethylation	167 (65.5)	66	57	72.28±3.17	1.00		1.00	
	Methylation	88 (34.5)	70	62	76.01±4.56	0.85 (0.57-1.27)	0.43	1.01 (0.66-1.54)	0.98
AOX-1	Unmethylation	116 (45.8)	64	58	72.75±3.84	1.00		1.00	
	Methylation	137 (54.2)	69	60	74.23±3.48	0.97 (0.67-1.41)	0.88	1.18 (0.79-1.77)	0.42
ADAMTS9	Unmethylation	154 (60.4)	66	58	73.04±3.29	1.00		1.00	
	Methylation	101 (39.6)	69	60	74.81±4.07	0.92 (0.63-1.35)	0.68	1.06 (0.70-1.60)	0.78
RERG	Unmethylation	142 (56.1)	67	58	73.35±3.38	1.00		1.00	
	Methylation	111 (43.9)	67	60	74.08±3.97	0.94 (0.64-1.37)	0.74	0.89 (0.59-1.33)	0.57
RARB2	Unmethylation	187 (73.3)	67	57	73.13±2.96	1.00		1.00	
	Methylation	68 (26.7)	68	65	69.53±5.14	0.90 (0.58-1.40)	0.63	0.91 (0.58-1.44)	0.69
MCSM	Non-MCSM	60 (27.9)	58	50	65.45±5.15	1.00		1.00	
	MCSM-L	65 (25.9)	73	63	78.03±4.87	0.68 (0.40-1.13)	0.14	1.04 (0.56-1.91)	0.91
	MCSM-H	126 (50.2)	68	61	74.25±3.71	0.74 (0.47-1.15)	0.18	1.04 (0.65-1.68)	0.87

MCSM	191 (76.1)	70	61	75.79±2.94	0.72 (0.47-1.09)	0.12	1.04 (0.66-1.65)	0.87

[a] Adjusted for Dukes staging, preoperative CA19-9 level and intestinal anastomosis

Table S5 Effects of combination and interaction between cereals intake and methylation of genes, MCSM on the risk of CRC

	Cereals			
	<100g /Week	≥100g /Week	Interaction	
	OR_{eg}[1] (95%CI)		OR_i (95%CI)	*P*
FOXE-1				
Unmethylation	1.00	0.48 (0.42-0.56)		
Methylation	1.26 (1.05-1.50)	0.79 (0.64-0.98)	1.31 (0.68-2.48)	0.42
IRF4				
Unmethylation	1.00	0.55 (0.48-0.63)		
Methylation	15.81 (8.27-30.23)	9.41 (3.65-24.23)	1.08 (0.81-14.35)	0.96
ADAMTS9				
Unmethylation	1.00	0.50 (0.43-0.58)		

Methylation	1.67 (1.41-1.97)	1.08 (0.87-1.33)	1.30 (0.69-2.44)	0.42
AOX1				
Unmethylation	1.00	0.41 (0.34-0.49)		
Methylation	1.38 (1.19-1.61)	1.03 (0.85-1.25)	1.82 (1.01-3.26)	0.045
RERG				
Unmethylation	1.00	0.49 (0.42-0.58)		
Methylation	1.76 (1.50-2.06)	1.28 (1.04-1.58)	1.50 (0.80-2.78)	0.20
RARB2				
Unmethylation	1.00	0.55 (0.47-0.63)		
Methylation	0.94 (0.80-1.12)	0.50 (0.39-0.62)	0.96 (0.49-1.88)	0.91
MCSM				
Unmethylation	1.00	0.37 (0.29-0.48)		
Methylation	1.26 (1.07-1.48)	0.80 (0.66-0.97)	1.72 (0.89-3.33)	0.11

[a] Adjusted for age, BMI, occupation and family history of cancer

Table S6 Effects of combination and interaction between vegetable intake and methylation of genes, MCSM on the risk of CRC

	Vegetable			
	<100g /Day	≥100g /Day	Interaction	
	OR_{eg}[1] (95%CI)		OR_i (95%CI)	*P*
FOXE-1				
Unmethylation	1.00	0.64 (0.49-0.84)		
Methylation	1.35 (0.84-2.18)	0.86 (0.65-1.14)	0.99 (0.32-3.02)	0.98
IRF4				
Unmethylation	1.00	0.62 (0.49-0.78)		
Methylation	-	10.26 (5.79-18.19)	-	-
ADAMTS9				
Unmethylation	1.00	0.78 (0.58-1.03)		
Methylation	2.37 (1.53-3.68)	1.36 (1.01-1.84)	0.75 (0.26-2.10)	0.58
AOX1				
Unmethylation	1.00	0.90 (0.67-1.21)		
Methylation	3.29 (2.11-5.14)	1.45 (1.07-1.96)	0.49 (0.17-1.39)	0.18
RERG				
Unmethylation	1.00	0.91 (0.69-1.22)		
Methylation	4.39 (2.74-7.02)	1.76 (1.31-2.37)	0.44 (0.15-1.33)	0.15
RARB2				
Unmethylation	1.00	0.71 (0.55-0.91)		
Methylation	1.56 (0.91-2.68)	0.66 (0.51-0.86)	0.60 (0.17-2.14)	0.43
MCSM				
Unmethylation	1.00	1.35 (0.89-2.06)		
Methylation	3.75 (2.32-6.05)	1.86 (1.23-2.81)	0.37 (0.12-1.14)	0.08

[a] Adjusted for age, BMI, occupation and family history of cancer

Table S7 Effects of combination and interaction between fat intake and methylation of genes, MCSM on the risk of CRC

	Fat			
	No	Yes	Interaction	
	OR_{eg}[1] (95%CI)		OR_i (95%CI)	*P*
FOXE-1				
Unmethylation	1.00	1.35 (1.18-1.55)		
Methylation	1.07 (0.88-1.31)	2.22 (1.82-2.70)	1.54 (0.82-2.92)	0.19
IRF4				
Unmethylation	1.00	1.50 (1.32-1.70)		
Methylation	21.69 (8.66-54.31)	20.64 (10.43-38.59)	0.62 (0.05-7.75)	0.71
ADAMTS9				
Unmethylation	1.00	1.60 (1.39-1.85)		
Methylation	1.83 (1.52-2.21)	2.95 (2.44-3.58)	1.01 (0.55-1.85)	0.99
AOX1				
Unmethylation	1.00	1.67 (1.42-1.96)		
Methylation	1.84 (1.55-2.19)	2.53 (2.13-3.04)	0.82 (0.48-1.43)	0.49
RERG				
Unmethylation	1.00	1.68 (1.45-1.96)		
Methylation	2.22 (1.86-2.66)	3.43 (2.83-4.15)	0.92 (0.51-1.64)	0.77
RARB2				
Unmethylation	1.00	1.65 (1.43-1.90)		
Methylation	1.07 (0.89-1.30)	1.43 (1.17-1.75)	0.81 (0.43-1.51)	0.51
MCSM				
Unmethylation	1.00	2.00 (1.59-2.51)		
Methylation	1.74 (1.44-2.10)	2.44 (2.03-2.95)	0.70 (0.38-1.29)	0.26

[a] Adjusted for age, BMI, occupation and family history of cancer

Table S8 Effects of combination and interaction between beef and mutton intake and methylation of genes, MCSM on the risk of CRC

	Beef and Mutton			
	＜250g /Week	⩾250g /Week	Interaction	
	OR_{eg}[1] (95%CI)		OR_i (95%CI)	*P*
FOXE-1				
Unmethylation	1.00	0.79 (0.66-0.96)		
Methylation	1.40 (1.20-1.63)	0.90 (0.66-1.23)	0.81 (0.34-1.93)	0.64
IRF4				
Unmethylation	1.00	0.73 (0.61-0.86)		
Methylation	14.09 (8.24-24.10)	-	-	-
ADAMTS9				
Unmethylation	1.00	0.86 (0.70-1.04)		
Methylation	2.07 (1.78-2.40)	0.99 (0.76-1.31)	0.56 (0.26-1.24)	0.15
AOX1				
Unmethylation	1.00	0.70 (0.56-0.88)		
Methylation	1.68 (1.47-1.93)	1.35 (1.05-1.72)	1.14 (0.53-2.43)	0.74
RERG				
Unmethylation	1.00	0.78 (0.64-0.96)		
Methylation	2.19 (1.90-2.53)	1.31 (1.01-1.71)	0.77 (0.36-1.65)	0.50
RARB2				
Unmethylation	1.00	0.88 (0.73-1.06)		
Methylation	1.08 (0.93-1.25)	0.40 (0.28-0.57)	0.42 (0.16-1.08)	0.07
MCSM				
Unmethylation	1.00	1.02 (0.74-1.41)		
Methylation	1.64 (1.42-1.90)	1.03 (0.84-1.29)	0.62 (0.27-1.42)	0.26

[a] Adjusted for age, BMI, occupation and family history of cancer

Table S9 Effects of combination and interaction between pork intake and methylation of genes, MCSM on the risk of CRC

	Pork			
	＜250g /Week	≥250g /Week	Interaction	
	OR_{eg}[1] (95%CI)		OR_i (95%CI)	*P*
FOXE-1				
Unmethylation	1.00	1.33 (1.16-1.53)		
Methylation	1.07 (0.89-1.30)	2.43 (1.97-2.99)	1.70 0.90-3.22)	0.10
IRF4				
Unmethylation	1.00	1.48 (1.31-1.680		
Methylation	28.98 (11.69-71.82)	16.10 (8.31-31.17)	0.37 (0.03-4.61)	0.44
ADAMTS9				
Unmethylation	1.00	1.64 (1.42-1.90)		
Methylation	2.14 (1.78-2.58)	2.44 (2.03-2.94)	0.70 (0.38-1.26)	0.23
AOX1				
Unmethylation	1.00	1.70 (1.44-1.99)		
Methylation	1.92 (1.62-2.27)	2.57 (2.15-3.08)	0.79 (0.45-1.39)	0.42
RERG				
Unmethylation	1.00	1.56 (1.34-1.81)		
Methylation	2.21 (1.85-2.63)	2.96 (2.46-3.56)	0.86 (0.48-1.53)	0.61
RARB2				
Unmethylation	1.00	1.45 (1.26-1.67)		
Methylation	0.91 (0.76-1.11)	1.48 (1.21-1.81)	1.12 (0.58-2.12)	0.74
MCSM				
Unmethylation	1.00	1.96 (1.56-2.46)		
Methylation	1.77 (1.47-2.13)	2.42 (2.00-2.93)	0.70 (0.38-1.28)	0.25

[a] Adjusted for age, BMI, occupation and family history of cancer

Table S10 Effects of combination and interaction between stewed fish with brown sauce intake and methylation of genes, MCSM on the risk of CRC

	Stewed fish with brown sauce			
	＜1 Times/Week	≥1 Times/Week	Interaction	
	OR_{eg}[1] (95%CI)		OR_i (95%CI)	*P*
FOXE-1				
Unmethylation	1.00	1.76 (1.50-2.07)		
Methylation	1.44 (1.22-1.69)	1.89 (1.50-2.40)	0.75 (0.37-1.50)	0.42
IRF4				
Unmethylation	1.00	1.67 (1.45-1.92)		
Methylation	22.67 (11.46-44.83)	14.97 (6.38-35.14)	0.40 (0.02-7.18)	0.53
ADAMTS9				
Unmethylation	1.00	2.06 (1.75-2.42)		
Methylation	2.25 (1.92-2.63)	2.31 (1.82-2.92)	0.50 (0.25-0.99)	0.046
AOX1				
Unmethylation	1.00	1.86 (1.55-2.23)		
Methylation	1.80 (1.55-2.08)	2.84 (2.31-3.48)	0.85 (0.45-1.58)	0.60
RERG				
Unmethylation	1.00	1.58 (1.33-1.87)		
Methylation	2.03 (1.75-2.36)	3.31 (2.66-4.12)	1.03 (0.54-1.97)	0.92
RARB2				
Unmethylation	1.00	1.59 (1.36-1.86)		
Methylation	0.95 (0.80-1.11)	1.65 (1.28-2.13)	1.10 (0.54-2.24)	0.79
MCSM				
Unmethylation	1.00	2.32 (1.80-2.99)		
Methylation	1.70 (1.45-1.99)	2.59 (2.13-3.16)	0.66 (0.33-1.31)	0.23

[a] Adjusted for age, BMI, occupation and family history of cancer

Table S11 Effects of combination and interaction between fried food intake and methylation of genes, MCSM on the risk of CRC

	Fried food			
	＜1 Times/Month	≥1 Times/Month	Interaction	
	OR_{eg}[1] (95%CI)		OR_i (95%CI)	*P*
FOXE-1				
Unmethylation	1.00	1.59 (1.35-1.87)		
Methylation	1.26 (1.08-1.48)	2.81 (2.14-3.70)	1.40 (0.65-3.02)	0.39
IRF4				
Unmethylation	1.00	1.78 (1.54-2.05)		
Methylation	15.18 (8.49-26.05)	-	-	-
ADAMTS9				
Unmethylation	1.00	1.85 (1.56-2.20)		
Methylation	1.94 (1.66-2.72)	2.67 (2.12-3.35)	0.74 (0.37-1.47)	0.39
AOX1				
Unmethylation	1.00	1.77 (1.48-2.12)		
Methylation	1.75 (1.52-2.01)	3.25 (2.59-4.07)	1.05 (0.54-2.02)	0.88
RERG				
Unmethylation	1.00	1.98 (1.67-2.34)		
Methylation	2.27 (1.96-2.63)	3.57 (2.78-4.58)	0.79 (0.39-1.60)	0.52
RARB2				
Unmethylation	1.00	1.93 (1.64-2.26)		
Methylation	1.08 (0.92-1.27)	1.31 (1.01-1.71)	0.63 (0.30-1.32)	0.22
MCSM				
Unmethylation	1.00	2.11 (1.64-2.72)		
Methylation	1.60 (1.37-1.88)	2.84 (2.32-3.47)	0.84 (0.42-1.68)	0.62

[a] Adjusted for age, BMI, occupation and family history of cancer

Table S12 Effects of combination and interaction between sausages intake and methylation of genes, MCSM on the risk of CRC

	Sausages			
	＜1 Times/Month	≥1 Times/Month	Interaction	
	OR_{eg}[1] (95%CI)		OR_i (95%CI)	*P*
FOXE-1				
Unmethylation	1.00	2.61 (2.17-3.13)		
Methylation	1.18 (1.01-1.38)	9.62 (6.36-14.56)	3.18 (0.88-11.45)	0.08
IRF4				
Unmethylation	1.00	3.24 (2.73-3.84)		
Methylation	15.54 (9.03-26.72)	-	-	-
ADAMTS9				
Unmethylation	1.00	3.38 (2.78-4.12)		
Methylation	1.89 (1.63-2.19)	5.12 (3.84-6.82)	0.80 (0.23-2.84)	0.72
AOX1				
Unmethylation	1.00	3.91 (3.12-4.90)		
Methylation	1.81 (1.57-2.08)	4.23 (3.35-5.35)	0.59 (0.25-1.39)	0.22
RERG				
Unmethylation	1.00	3.89 (3.17-4.78)		
Methylation	2.28 (1.97-2.63)	4.74 (3.66-6.16)	0.54 (0.22-1.29)	0.16
RARB2				
Unmethylation	1.00	3.37 (2.79-4.08)		
Methylation	0.995 (0.85-1.16)	2.71 (1.99-3.68)	0.80 (0.29-2.26)	0.67
MCSM				
Unmethylation	1.00	5.53 (4.08-7.49)		
Methylation	1.75 (1.50-2.05)	4.47 (3.59-5.58)	0.46 (0.18-1.15)	0.10

[a] Adjusted for age, BMI, occupation and family history of cancer

Table S13 Effects of combination and interaction between pungent food intake and methylation of genes, MCSM on the risk of CRC

	Pungent food			
	＜4 Times/Week	≥4 Times/Week	Interaction	
	OR_{eg}[1] (95%CI)		OR_i (95%CI)	*P*
FOXE-1				
Unmethylation	1.00	0.74 (0.65-0.86)		
Methylation	1.23 (1.02-1.48)	1.10 (0.90-1.36)	1.21 (0.64-2.28)	0.56
IRF4				
Unmethylation	1.00	0.75 (0.67-0.85)		
Methylation	26.74 (10.85-65.94)	8.99 (4.60-17.57)	0.45 (0.04-5.54)	0.53
ADAMTS9				
Unmethylation	1.00	0.77 (0.67-0.89)		
Methylation	1.74 (1.46-2.08)	1.47 (1.21-1.79)	1.10 (0.60-2.02)	0.76
AOX1				
Unmethylation	1.00	0.73 (0.62-0.86)		
Methylation	1.59 (1.34-1.88)	1.36 (1.14-1.63)	1.17 (0.68-2.03)	0.57
RERG				
Unmethylation	1.00	0.84 (0.72-0.97)		
Methylation	2.28 (1.91-2.73)	1.55 (1.29-1.86)	0.81 (0.45-1.45)	0.48
RARB2				
Unmethylation	1.00	0.90 (0.78-1.03)		
Methylation	1.30 (1.08-1.58)	0.61 (0.50-0.75)	0.52 (0.28-0.99)	0.047
MCSM				
Unmethylation	1.00	0.68 (0.55-0.85)		
Methylation	1.38 (1.15-1.67)	1.12 (0.93-1.35)	1.18 (0.65-2.16)	0.58

[a] Adjusted for age, BMI, occupation and family history of cancer

Table S14 Effects of combination and interaction between sauerkraut intake and methylation of genes, MCSM on the risk of CRC

	Sauerkraut			
	＜3 Times/Week	≥3 Times/Week	Interaction	
	OR_{eg}[1] (95%CI)		OR_i (95%CI)	*P*
FOXE-1				
Unmethylation	1.00	0.67 (0.58-0.78)		
Methylation	1.74 (1.37-2.21)	0.80 (0.66-0.97)	0.68 (0.35-1.34)	0.27
IRF4				
Unmethylation	1.00	0.58 (0.51-0.65)		
Methylation	13.41 (5.35-33.62)	11.12 (5.77-21.44)	1.44 (0.12-18.05)	0.78
ADAMTS9				
Unmethylation	1.00	0.66 (0.56-0.76)		
Methylation	2.09 (1.68-2.61)	1.09 (0.91-1.32)	0.80 (0.43-1.49)	0.48
AOX1				
Unmethylation	1.00	0.67 (0.57-0.80)		
Methylation	1.98 (1.61-2.43)	1.07 (0.90-1.28)	0.81 (0.45-1.43)	0.46
RERG				
Unmethylation	1.00	0.67 (0.57-0.78)		
Methylation	2.46 (1.98-3.06)	1.27 (1.06-1.52)	0.77 (0.42-1.42)	0.40
RARB2				
Unmethylation	1.00	0.55 (0.48-0.64)		
Methylation	0.76 (0.61-0.95)	0.60 (0.49-0.72)	1.41 (0.74-2.70)	0.30
MCSM				
Unmethylation	1.00	0.65 (0.52-0.82)		
Methylation	1.59 (1.28-1.98)	0.97 (0.79-1.18)	0.93 (0.50-1.73)	0.82

[a] Adjusted for age, BMI, occupation and family history of cancer

Table S15 Effects of combination and interaction between overnight food intake and methylation of genes, MCSM on the risk of CRC

	Overnight food			
	＜3 Times/Week	≥3 Times/Week	Interaction	
	OR_{eg}[1] (95%CI)		OR_i (95%CI)	*P*
FOXE-1				
Unmethylation	1.00	1.55 (1.35-1.79)		
Methylation	1.30 (1.09-1.55)	2.48 (1.97-3.12)	1.23 (0.64-2.36)	0.54
IRF4				
Unmethylation	1.00	1.80 (1.58-2.04)		
Methylation	44.89 (18.28-110.28)	9.60 (4.83-19.10)	0.12 (0.01-1.50)	0.10
ADAMTS9				
Unmethylation	1.00	1.64 (1.41-1.90)		
Methylation	1.83 (1.54-2.17)	3.00 (2.45-3.68)	1.01 (0.54-1.85)	0.99
AOX1				
Unmethylation	1.00	1.65 (1.40-1.95)		
Methylation	1.72 (1.47-2.02)	3.04 (2.51-3.68)	1.07 (0.61-1.88)	0.82
RERG				
Unmethylation	1.00	1.74 (1.49-2.03)		
Methylation	2.17 (1.84-2.56)	3.32 (2.74-4.04)	0.88 (0.48-1.60)	0.68
RARB2				
Unmethylation	1.00	1.98 (1.71-2.29)		
Methylation	1.29 (1.08-1.53)	1.22 (0.97-1.53)	0.48 (0.25-0.93)	0.03
MCSM				
Unmethylation	1.00	1.17 (0.93-1.48)		
Methylation	1.24 (1.04-1.47)	2.48 (2.05-3.01)	1.71 (0.92-3.18)	0.09

[a] Adjusted for age, BMI, occupation and family history of cancer

Table S16 Effects of combination and interaction between smoking and methylation of genes, MCSM on the risk of CRC

	Smoking			
	No	Yes	Interaction	
	OR_{eg}[1] (95%CI)		OR_i (95%CI)	*P*
FOXE-1				
Unmethylation	1.00	1.42 (1.12-1.81)		
Methylation	0.92 (0.77-1.11)	0.79 (0.65-0.96)	1.66 (0.89-3.11)	0.11
IRF4				
Unmethylation	1.00	-		
Methylation	0.11 (0.06-0.19)	0.11 (0.06-0.19)	-	-
ADAMTS9				
Unmethylation	1.00	0.88 (0.71-1.11)		
Methylation	0.52 (0.43-0.62)	0.51 (0.42-0.62)	0.89 (0.49-1.63)	0.71
AOX1				
Unmethylation	1.00	1.19 (0.99-1.44)		
Methylation	0.66 (0.56-0.78)	0.58 (0.49-0.69)	1.35 (0.77-2.37)	0.29
RERG				
Unmethylation	1.00	1.49 (1.21-1.85)		
Methylation	0.62 (0.53-0.74)	0.51 (0.42-0.60)	1.84 (1.02-3.31)	0.04
RARB2				
Unmethylation	1.00	0.64 (0.50-0.81)		
Methylation	0.81 (0.68-0.98)	0.92 (0.76-1.11)	0.56 (0.30-1.06)	0.07
MCSM				
Unmethylation	1.00	0.78 (0.62-0.97)		
Methylation	1.28 (1.07-1.53)	1.44 (1.20-1.73)	1.45 (0.79-2.66)	0.23

[a] Adjusted for age, BMI, occupation and family history of cancer

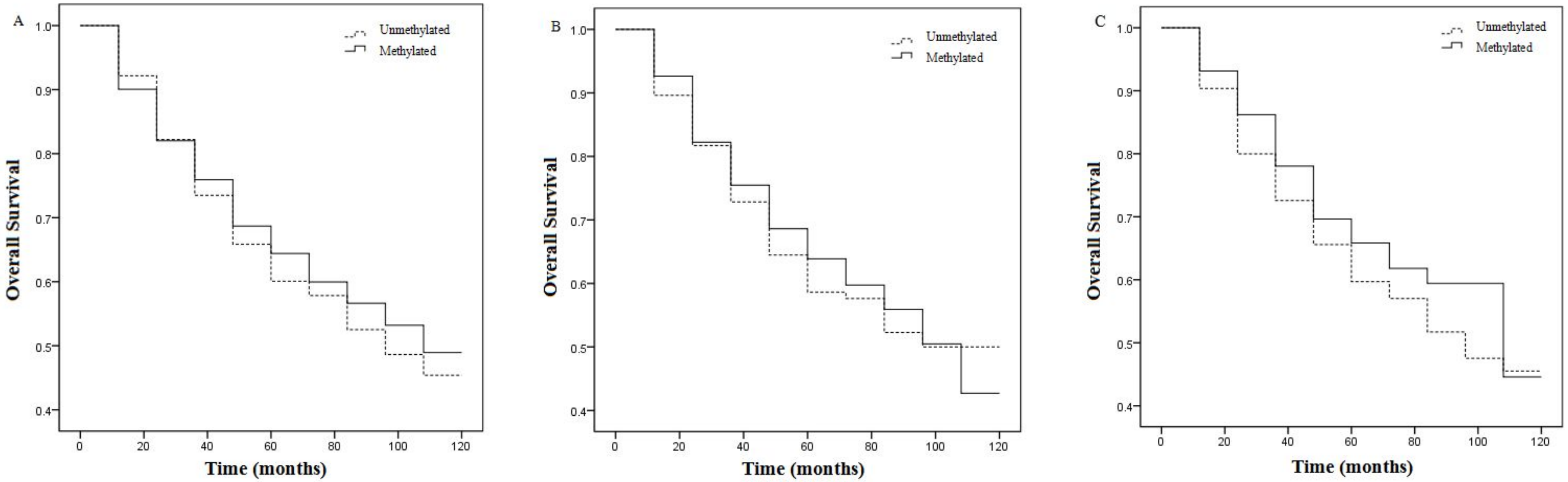
A
B
C
Unmethylated
Methylated
Overall Survival
Time (months)
1.0
0.9
0.8
0.7
0.6
0.5
0.4
0
20
40
60
80
100
120

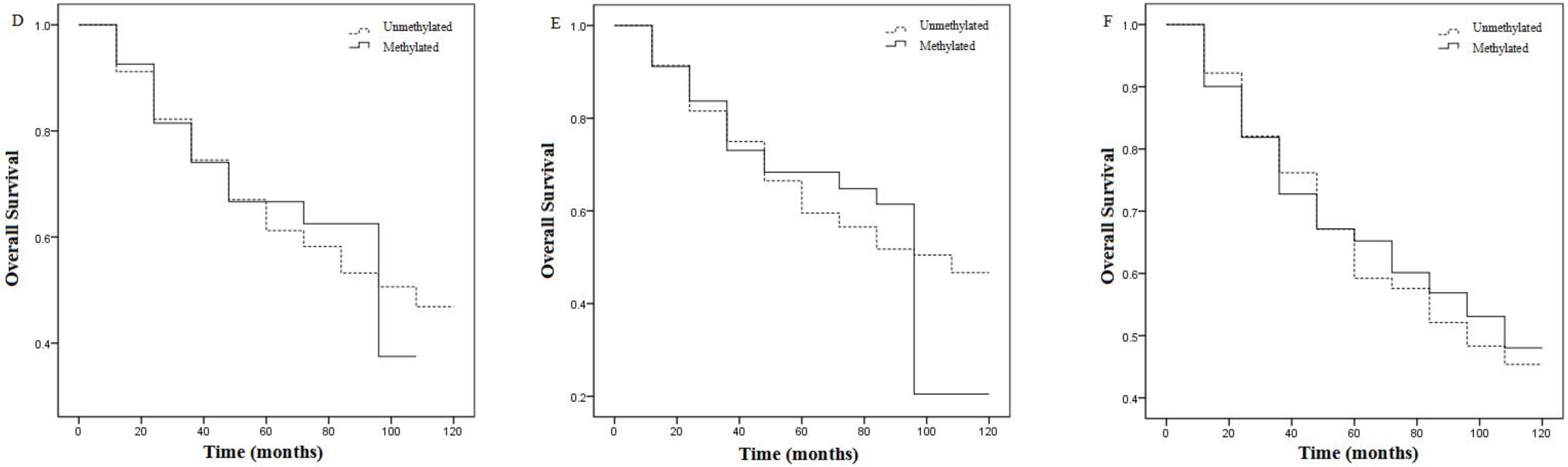
D
E
F
Unmethylated
Methylated
Overall Survival
Time (months)
1.0
0.9
0.8
0.7
0.6
0.5
0.4
0.2
0
20
40
60
80
100
120

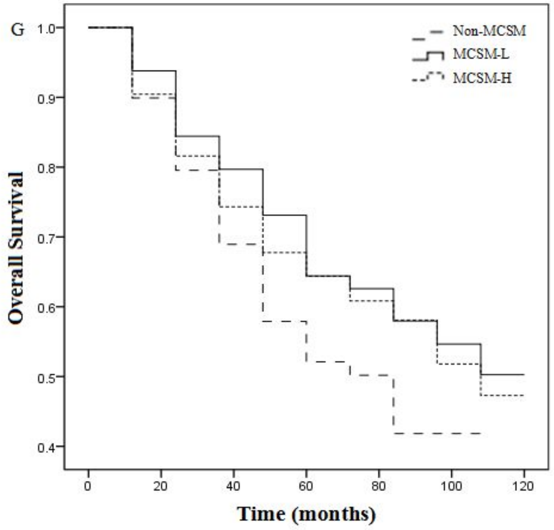

Figure S1 A-G Kaplan-Meier curves for cumulative survival comparisons between patients with unrmethylated and methylated of ADAMST9, AOX-1, FOXE-1, IRF4, RARB2, RERG and MCSM, respectively
